# SCOPING REVIEW OF CLINICAL STUDIES EXPLORING THE ASSOCIATION BETWEEN EPIGENETIC AGE AND FRAILTY

**DOI:** 10.1093/geroni/igad104.3582

**Published:** 2023-12-21

**Authors:** Ebba Hillstedt, Rebecka Rubsenson Wahlin, Markus Castegren, Gabriel Sandblom, Alice Kane

**Affiliations:** Karolinska institutet, Stockholm, Stockholms Lan, Sweden; Karolinska institutet, Stockholm, Stockholms Lan, Sweden; Karolinska institutet, Stockholm, Stockholms Lan, Sweden; Karolinska institutet, Stockholm, Stockholms Lan, Sweden; Institute for Systems Biology, Seattle, Washington, United States

## Abstract

An overall increase in life expectancy shines light on the complexity of human aging where some individuals seem to age at a faster pace than others, a phenomenon health care systems are already battling to identify. Multiple frailty scoring systems have been proposed as models for biological age estimations but their implementation within health care systems is time consuming and often deprioritized. Epigenetic clocks based on DNA methylation (DNAm) changes have been proposed as newer estimations of biological age, but there is conflicting data on the correlation between these two measures. We conducted a review of the literature to identify studies that have explored the correlation between frailty and epigenetic age. Our search strategy identified 37 articles, and after further screening we found 15 articles that directly compared frailty scores to epigenetic age. Most studies measured DNAm in whole blood (9/15), although there was great variability in the specific epigenetic clocks applied. Almost all studies measured frailty with either Fried’s frailty phenotype (4/15) or a Frailty Index (9/15). The studies were divided in their findings, with 7/15 showing moderate to strong associations and the other 8 studies showing no or weak associations. Interestingly, the associations with frailty were usually seen for epigenetic clocks predicting mortality or other phenotypes (eg GrimAge, PhenoAge), rather than epigenetic clocks predicting chronological age. More work is needed to understand the relationship between these biological age measures and their potential role in clinical risk assessment for older adults.

